# Lack of association between cathepsin D C224T polymorphism and Alzheimer’s disease risk: an update meta-analysis

**DOI:** 10.1186/1471-2377-14-13

**Published:** 2014-01-15

**Authors:** Cuiju Mo, Qiliu Peng, Jingzhe Sui, Jian Wang, Yan Deng, Li Xie, Taijie Li, Yu He, Xue Qin, Shan Li

**Affiliations:** 1 Department of Clinical Laboratory, First Affiliated Hospital of Guangxi Medical University, Nanning, Guangxi 530021, China

**Keywords:** Cathepsin D, AD, Polymorphism, Meta-analysis

## Abstract

**Background:**

Cathepsin D C224T polymorphism has been reported to associate with AD susceptibility. But the results were inconsistent. This study aimed to assess the relationship between C224T polymorphism and AD risk.

**Methods:**

The relevant studies were identified by searching PubMed, Embase, Web of Science, Google Scholar and Wan fang electronic databases updated on July 2013. The relationship between Cathepsin D C224T polymorphism and AD risk was evaluated by ORs and 95% CIs.

**Results:**

A total of 25 case-control studies including 5,602 cases and 11,049 controls were included in the meta-analysis. There was no association between C224T polymorphism and AD risk with all the studies were pooled in the meta-analysis (CT vs. CC: OR = 1.125, 95% CI = 0.974-1.299, P = 0.109; CT + TT vs. CC: OR = 1.136, 95% CI = 0.978-1.320, P = 0.094). Furthermore, when stratified by ethnicity, age of onset and APOEϵ4 status, significant association did not found in all subgroups.

**Conclusion:**

The present meta-analysis suggested that the Cathepsin D C224T polymorphism was not associated with AD susceptibility.

## Background

The neurodegenerative disorder Alzheimer’s disease (AD) caused the most of dementia in the elderly [1]. Previous findings indicated that the incidence increased from 1% in 65–69 year-olds to about 50% in 85–95 year-olds [2]. Many genetic and environmental risk factors contribute to the degenerative progress of AD, such as family history, low income and education, exposure to aluminium in drinking water, dietary habits, smoking, physical activity, hypertension, diabetes and genetic variations [3]. Molecular genetics researches have shown that AD was a class of complex polygenic diseases with genetic heterogeneity. Several genes have been reported to associate with AD. Beta-amyloid precursor protein (APP) and presenilin 2 played major role in early-onset familial AD [4,5]. The death-associated protein kinase 1(DAPK1) [6] and ATP-binding cassette subfamily A member 7 (ABCA7) [7] have been mainly implicated with late-onset AD. The ϵ4 allele of apolipoprotein E (*APOEϵ4*) was the only verified risk factor for sporadic AD [8]. However, the presence of variants for these genes and the *APOEϵ4* allele was neither necessary nor sufficient for AD development. About 50% of AD patients did not have mutations in the genes mentioned above or carry the *APOEϵ4* allele, and not everyone who has the mutations of the genes will acquire AD [9], suggesting that it is necessary to identify additional genetic or non-genetic factors which modulate the AD susceptibility.

The main histopathologic features of AD are neurofibrillary tangles and Neuritic plaques which consist of hyperphosphorylated tau protein and amyloid peptides, respectively. Cathepsin D (*CTSD*), an intracellular acid protease, contributed to the proteolytic cleavage of APP and the clearance of the β-amyloid (Aβ) from the central nervous system [10,11]. As such, *CTSD* might involve in the pathogenesis of AD. Variants of *CTSD* gene might impede the functions of proteolytic degradation, thus increasing the risk of AD. A *CTSD C224T* polymorphism(C-to-T) in exon 2 can bring about amino acid change (Ala38-to-Val), increase pro-CTSD secretion and alter intracellular maturation [12]. It has been proved that this polymorphism was significantly associated with the general intelligence of healthy elderly [13].

Recently, numerous studies have focused on the correlation between the *CTSD C224T* polymorphism and AD risk [14-36]. Unfortunately, the results of these studies were contradictory. Five previous studies reported that the *T* allele of the *CTSD-C/T* polymorphism was a high-risk factor for developing AD [14-18]; however, other relevant studies yielded contradictory results [19-36]. Furthermore, the results of previous meta-analysis which research the association between the *CTSD* polymorphism and AD risk were contradictory as well. Bertram et al. [37] and Ntais et al. [38] did not find any significant association, whereas Schuur [18] reported that *T* allele increased the risk of AD in Caucasians. Possible reasons for these contradictory results include the small sample size of the Ntais study; the absence of an Asian population in the Schuur study; and the fact that the Bertram study only compared alleles *T* and *C*. Considering that those factors could contribute to bias in the final result, we updated the present meta-analysis which included a larger sample size to provide a more reliable correlation between *CTSD C224T* and AD.

## Methods

### Search strategy

The relevant studies were identified by searching PubMed, Embase, Web of Science, Google Scholar and Wan fang electronic databases in July 2013 for all the articles regarding the correlation between *CTSD C224T* polymorphism and AD risk. The key words of search strategy as follow: “Alzheimer’s disease or AD”, “CTSD or cathepsin D”, and “polymorphism, mutation or variant”. References listed in reviews and retrieved articles were also screened. There were no language or country restrictions. When multiple articles researched the same cohort, the one with the largest population was included. When a publication reported more than one subpopulation, we regarded every subpopulation as a separate study.

### Selection criteria

The eligible studies were requested to agree with the inclusion criteria: (1) a case–control study; (2) research of the correlation between *CTSD C224T* polymorphism and AD susceptibility; (3)inclusion of the sample size and distribution of alleles and genotypes; (4) AD diagnosed according to the criteria of the National Institute of Neurological and Communicative Disorders and Stroke and the Alzheimer’s Disease and Related Disorders Association (NINCDS-ADRDA), or the Diagnostic and Statistical Manual of Mental Disorders, Fourth Edition (DSM-IV). Exclusion criteria of our study were followed as: (1) duplicated literature, reviews, or animal studies; (2) genotype frequency and distribution were not included; (3) not enough information for data extraction.

### Data extraction

Two reviewers (Cuiju Mo and Jingzhe Sui) extracted the information independently. If there was a disagreement, the data was checked again, and a third reviewer (Xue Qin) was invited to check the data. Information collected from each eligible study was included: first author, year of publication, country, ethnicity, genotyping method, AD diagnosis, control sources, sample sizes, age of onset, and genotype distribution in cases and controls.

### Statistical analysis

All analysis was conducted using Stata version 12.0 software (Stata Corp, College Station, TX). The association was assessed by pooled odds ratio (OR) together with the corresponding 95% confidence interval (CI). Only heterozygote comparison model (TC vs. CC) and the dominant genetic model (TT + TC vs. CC) were analysed. Furthermore, we evaluated the effect in different subgroup stratified by ethnicity (Asian vs. Caucasian) and age of onset. Early-onset AD (EOAD) was defined as age at onset <65 years, and age at onset ≥65 years was considered as late-onset AD (LOAD). To evaluate the interaction of the *CTSD* with the *APOEϵ4* allele, we compared the dominant genetic model (TT + TC vs. CC) between case and control subjects stratified by the *APOEϵ4* allele status. Similarly, the relationship of the *APOEϵ4* allele with AD risk was investigated between the patients carrying the *T* allele or not.

The x^2^-test based Q-statistic and I^2^ statistic was used to evaluate the heterogeneity among the studies. The DerSimonian–Laird random-effects model was used to assess pooled OR when a significant heterogeneity (P_Q_ < 0.1 or I^2^ ≥ 50%) was observed. Otherwise, the Mantel–Haenszel fixed-effects model was used. The publication bias was detected by funnel plot and Egger’s test. An Egger’s test P value <0.05 was considered as statistically significant. The genotype distribution of the control population was used to evaluate Hardy–Weinberg Equilibrium (HWE) by a goodness-of-fit Chi-square test. P <0.05 (two-side) was considered as statistically significant.

## Results

### Eligible studies

**Figure 1:**
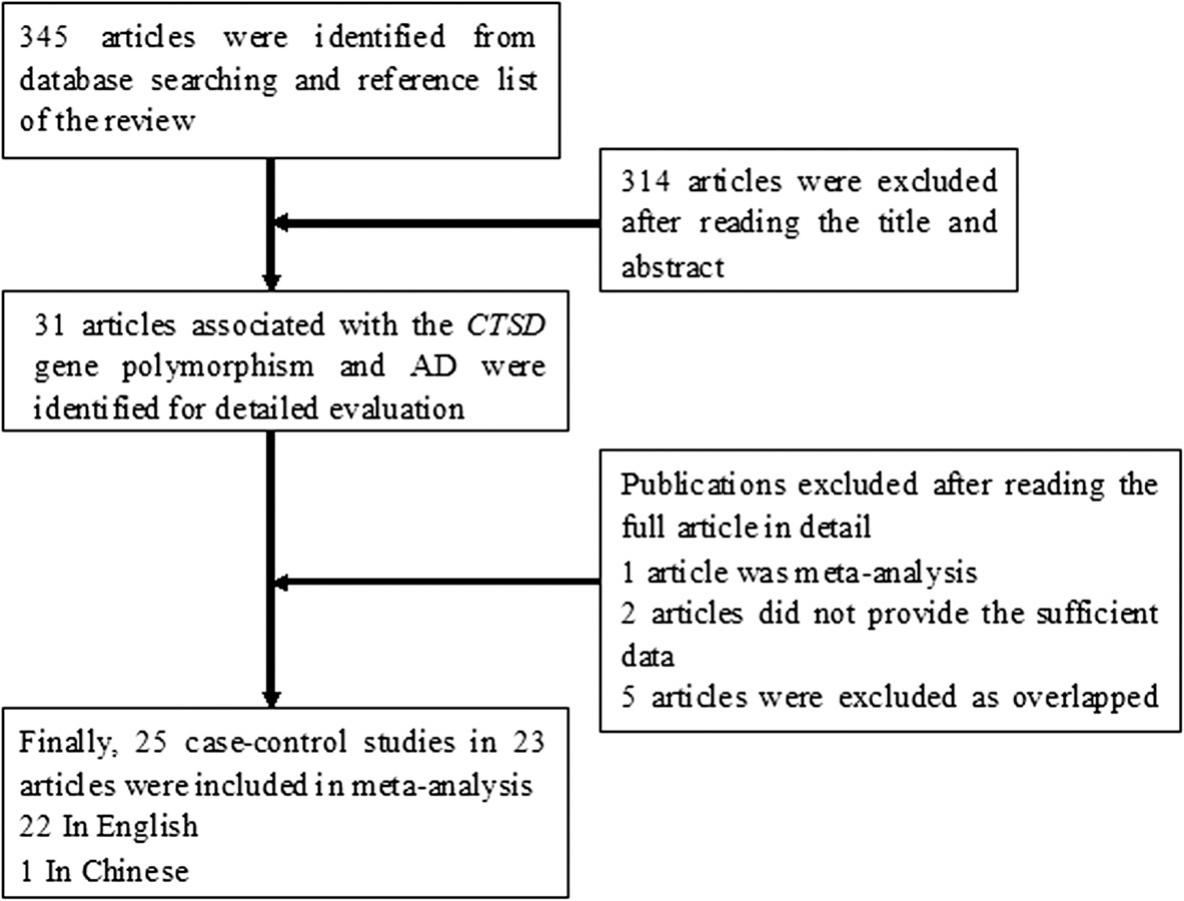
Flow chart of literature screening for this meta-analysis.

Figure 1 showed the screening process of our study. A total of 345 articles were identified from the database searching and references of review. 31 relevant articles were identified according to inclusion criteria. Then eight articles were excluded based on the full texts: one article was a meta-analysis [38], two articles did not provide sufficient data [39,40], and five articles overlapped with other published studies [32,41-44]. Finally, 23 articles including 22 English papers and 1 Chinese paper [19] were included in our study. Two out of the including articles reported two subpopulations, and each subpopulation was considered as a separate study. Therefore, 25 case–control studies including 5,602 cases and 11,049 controls were included in the meta-analysis, encompassing 4 Asian and 21 Caucasian samples. All AD patients were diagnosed by NINCDS-ADRDA criteria, DSM-IV criteria, or autopsy confirmation in all eligible studies. The genotype frequencies of the control groups in two case–control studies deviated from the HWE [22,29]. Ten of the eligible studies evaluated the interaction between the *CTSD* and the *APOEϵ4* allele [1,14,15,18-21,24,28,29,34]. Six of the studies included early-onset and late-onset cases [20,21,28,29,33,34]. The baseline data of each case–control study were presented in Table 1.

**Table 1:** The baseline data of all including study

**First author**	**Year**	**Country**	**Ethnicity**	**Genotyping method**	**AD diagnostic**	**Control sources**	**HWE**	**Case (EOAD/LOAD)**	**Control**
Sun	2005	China	Asian	PCR-RFLP	NINCDS-ADRDA and DSM-IV	PB	0.552	165	174
Li	2004	China	Asian	PCR-RFLP	NINCDS-ADRDA	PB	0.484	156(42/114)	183
Jhoo	2005	Korea	Asian	DASH	NINCDS-ADRDA	PB	0.701	107(36/71)	216
Matsui	2001	Japan	Asian	PCR-RFLP	NINCDS-ADRDA	PB	0.000	275	479
		USA	Caucasian	PCR-RFLP	autopsy-confirmed	PB	0.191	69	50
Papassotiropoulos	1999	Germany	Caucasian	PCR-RFLP	NINCDS-ADRDA	PB	0.21	102	351
McIlroy	1999	Ireland	Caucasian	PCR-RFLP	DSM IV and NINCDS-ADRDA	PB	0.367	183	187
Papassotiropoulos	2000(b)	Germany	Caucasian	PCR-RFLP	NINCDS-ADRDA	HB	0.485	127	184
Bhojak	2000	USA	Caucasian	PCR-RFLP	NINCDS-ADRDA	HB	0.084	531	316
Crawford	2000	USA	Caucasian	PCR-RFLP	NINCDS-ADRDA	HB	0.319	210	120
		Spain	Caucasian	PCR-RFLP	NINCDS-ADRDA	HB	0.101	79	112
Menzer	2001	Germany, Switzerland, Italy	Caucasian	PCR-RFLP	NINCDS-ADRDA	HB and PB	0.988	324	302
Bertram	2001	USA	Caucasian	PCR-RFLP	NINCDS-ADRDA	HB	0.373	200	182
Emahazion	2001	Scotland	Caucasian	DASH	DSM-IV	Not clarified.	0.329	120	149
Bagnoli	2002	Italy	Caucasian	PCR-RFLP	DSM-IV	PB	0.616	197(33/33)	126
Mateo	2002	Spain	Caucasian	PCR-RFLP	NINCDS-ADRDA	HB	0.008	311(126/185)	346
Styczynska	2003	Polish	Caucasian	PCR-RFLP	NINCDS-ADRDA	HB	0.637	100	100
Ingegni	2003	Italy	Caucasian	PCR-RFLP	NINCDS-ADRDA	HB	0.914	142	120
Beryer	2005	Spain	Caucasian	PCR-RFLP	DSM-IV and NINCDS-ADRDA	Not clarified.	0.871	205	181
Blomqvist2	2006	Switzerland	Caucasian	DASH	NINCDS-ADRDA	HB and PB	0.372	385	173
Mariani	2006	Italy	Caucasian	PCR-RFLP	NINCDS-ADRDA	PB	0.355	100	136
Davidson	2006	UK	Caucasian	PCR-RFLP	NINCDS-ADRDA	HB	0.168	560(317/243)	767
Capurso	2008	Italy	Caucasian	PCR-RFLP	NINCDS-ADRDA	PB	0.205	242(57/185)	421
Albayrak	2010	Germany	Caucasian	PCR-RFLP	NINCDS-ADRDA	HB	0.143	219	215
M. Schuur	2011	Netherland	Caucasian	Taqman assay	NINCDS-ADRDA	PB	0.631	493	5619

*PCR–RFLP*, Polymerase chain reaction-restriction fragment length polymorphism; *DASH*, dynamic allele specific hybridization; *PB*, Population–based; *HB*, Hospital–based; *HWE*, hardy-Weinberg equilibrium; *EOAD*, early-onset AD; *LOAD*, late-onset AD.

### Results of meta-analysis

**Figure 2:**
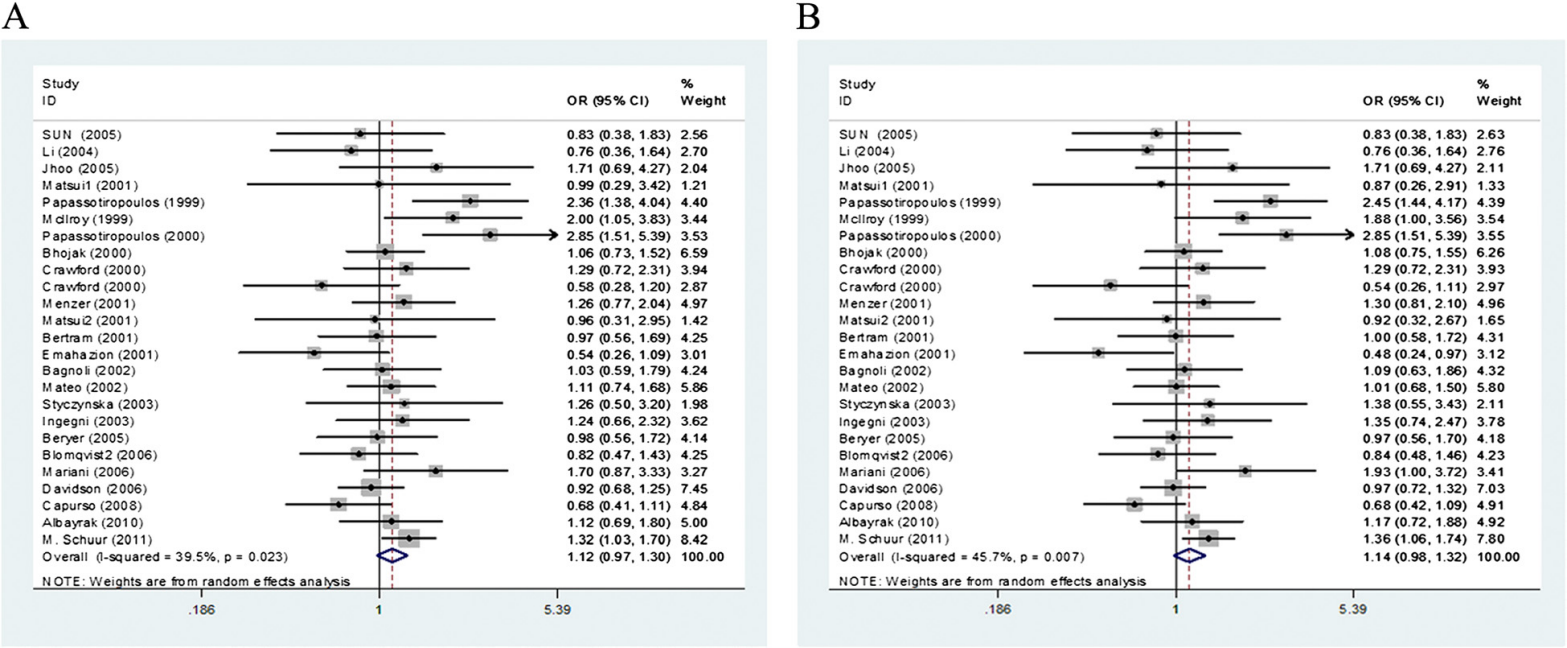
**Forest plots of *CTSD C224T *** **polymorphism and AD risk (A, CT vs. CC model; B, TT + CT vs. CC model) in all analysis using random-effect model.**

The present finding of this meta-analysis revealed that the *C224T* polymorphism was not associated with AD risk. The heterogeneities of CT vs. CC and the dominant CT + TT vs. CC models were assessed in the overall population, and the P_Q_ values were 0.023 and 0.007, respectively. Thus, random-effects model was chose to analyse the CT vs. CC model (OR = 1.125, 95% CI = 0.974–1.299, P = 0.109, Table 2, Figure 2A) and the dominant CT + TT vs. CC model (OR = 1.136, 95% CI = 0.978–1.320, P = 0.094, Table 2, Figure 2B) in the overall population. The control genotypes of two case-control studies [22,29] deviated from the HWE. The summary ORs were slightly elevated in the CT vs. CC (OR = 1.127, 95% CI = 0.965-1.317, P = 0.132) and dominant CT + TT vs. CC models (OR = 1.149, 95% CI = 0.978-1.35, P = 0.09) without a statistical significance, when we excluded those two studies.

**Table 2:** **Results of the association between *CTSD C224T *** **polymorphism and AD risk in the meta-analysis**

**Comparison**	**Population**	**No. of studies**	**Test of association**			**Mode**	**Test of heterogeneity**		
			**OR**	**95% CI**	**P Value**		***x***^**2**^	**P**_**Q **_**Value**	**I**^**2**^
CT vs. CC	Overall	25	1.125	0.974–1.299	0.109	R	39.65	0.023	39.5
CT + TT vs. CC	Overall	25	1.136	0.978–1.320	0.094	R	44.23	0.007	45.7
Subgroup analysis									
Ethnicity									
CT vs. CC	Asian	4	0.971	0.626–1.506	0.895	F	2.04	0.565	0.0
	Caucasian	21	1.139	0.974–1.331	0.102	R	37.20	0.011	46.2
CT + TT vs. CC	Asian	4	0.954	0.616–1.477	0.833	F	2.04	0.565	0.0
	Caucasian	21	1.154	0.982–1.357	0.082	R	41.54	0.003	51.8
EOAD									
CT vs. CC	Overall	6	0.937	0.706–1.245	0.654	F	2.87	0.719	0.0
CT + TT vs. CC	Overall	6	0.93	0.704–1.229	0.612	F	2.68	0.749	0.0
LOAD									
CT vs. CC	Overall	6	0.935	0.724–1.207	0.606	F	3.86	0.57	0.0
CT + TT vs. CC	Overall	6	0.931	0.726–1.195	0.575	F	3.88	0.567	0.0

*OR*, odds ratio; *CI*, confidence intervals; *R*, random effects model; *F*, fixed effects model; *EOAD*, early-onset AD; *LOAD* ,late-onset AD.

In subgroup analyses stratified by ethnicity, we failed to find any significant associations between the *CTSD C224T* polymorphism and AD risk in the Asian (CT vs. CC: OR = 0.971, 95% CI = 0.626-1.506, P = 0.895; CT + TT vs. CC: OR = 0.954, 95% CI = 0.616-1.477, P = 0.833, Table 2) and Caucasian(CT vs. CC: OR = 1.139, 95% CI = 0.974-1.331, P = 0.102; CT + TT vs. CC: OR = 1.154, 95% CI = 0.982-1.357, P = 0.082, Table 2) populations. After excluding two studies [22,29] which deviated from the HWE,no significant associations were found between the *CTSD C224T* polymorphism and AD risk in the Asian (CT + TT vs. CC: OR = 0.968, 95% CI =0.605-1.548, P = 0.891) and Caucasian(CT + TT vs. CC: OR = 1.165, 95% CI =0.981-1.383, P = 0.081). Similarly, we found non-significant associations in the EOAD (CT vs. CC: OR = 0.937, 95% CI = 0.706-1.245, P = 0.654; CT + TT vs. CC: OR = 0.930, 95% CI = 0.704-1.229, P = 0.612) and LOAD (CT vs. CC: OR = 0.935, 95% CI = 0.724-1.207, P = 0.606; CT + TT vs. CC: OR = 0.931, 95% CI = 0.726-1.195, P = 0.575)subgroups in any of the comparisons (Table 2).

In the *APOEϵ4* stratified analyses, the results did not show significant associations between the *C224T* polymorphism and AD risk in *APOEϵ4* carriers and non-carriers. However, the pooled OR were higher in *APOEϵ4* carriers (CT + TT vs. CC: OR = 1.267, 95% CI = 0.979-1.641, P = 0.072, Table 3) than in non-carriers (CT + TT vs. CC: OR = 1.139, 95% CI = 0.844-1.539, P = 0.395, Table 3). Furthermore, among the *T* allele carriers, *APOEϵ4* allele increased the risk of AD 4.5-fold (OR = 4.532, 95% CI = 2.755-7.455, P = 0.000, Table 3) accompanied by heterogeneity (P = 0.033). Among the subjects without the *T* allele, *APOEϵ4* increased the risk of AD 4.2-fold (OR = 4.193, 95% CI =3.096-5.679, P = 0.000, Table 3) with significant between-study heterogeneity (P = 0.000). Extensive overlap existed between the two estimates; however, the ORs were greater in the *T* allele carriers.

**Table 3:** **Meta-analysis the association of *CTSD C224T *** **polymorphism with *APOEϵ4 *** **carrier in AD**

**Comparison**	**Population**	**No. of studies**	**Test of association**			**Mode**	**Test of heterogeneity**		
			**OR**	**95% CI**	**P Value**		***x***^**2**^	**PQ Value**	**I**^**2**^
APOEϵ4 noncarriers									
CT + TT vs. CC	Overall	10	1.139	0.844–1.539	0.395	R	19.28	0.023	53.3
	Asian	3	0.73	0.390–1.365	0.324	F	5.81	0.055	65.5
	Caucasian	7	1.212	0.998–1.472	0.052	F	11.86	0.065	49.4
APOEϵ4 carriers									
CT + TT vs. CC	Overall	10	1.267	0.979–1.641	0.072	F	10.89	0.283	17.4
	Asian	3	1.273	0.511–3.184	0.604	F	0.01	0.995	0.0
T carriers	Caucasian	7	1.267	0.979–1.641	0.085	F	10.88	0.092	44.9
APOEϵ4(+) vs. APOEϵ4(–)	Overall	10	4.532	2.755–7.455	0.000	R	18.16	0.033	50.4
	Asian	3	7.913	2.632–23.785	0.000	F	0.20	0.904	0.0
	Caucasian	7	4.134	2.338–7.310	0.000	R	15.58	0.016	61.5
T noncarriers									
APOEϵ4(+) vs. APOEϵ4(–)	Overall	10	4.193	3.096–5.679	0.000	R	43.54	0.000	79.3
	Asian	3	4.217	2.333–7.620	0.000	R	6.88	0.032	70.9
	Caucasian	7	4.195	2.888–6.093	0.000	R	35.89	0.000	83.3

*OR*, odds ratio; *CI*, confidence intervals; *R*, random effects model; *F*, fixed effects model.

### Publication bias

**Figure 3:**
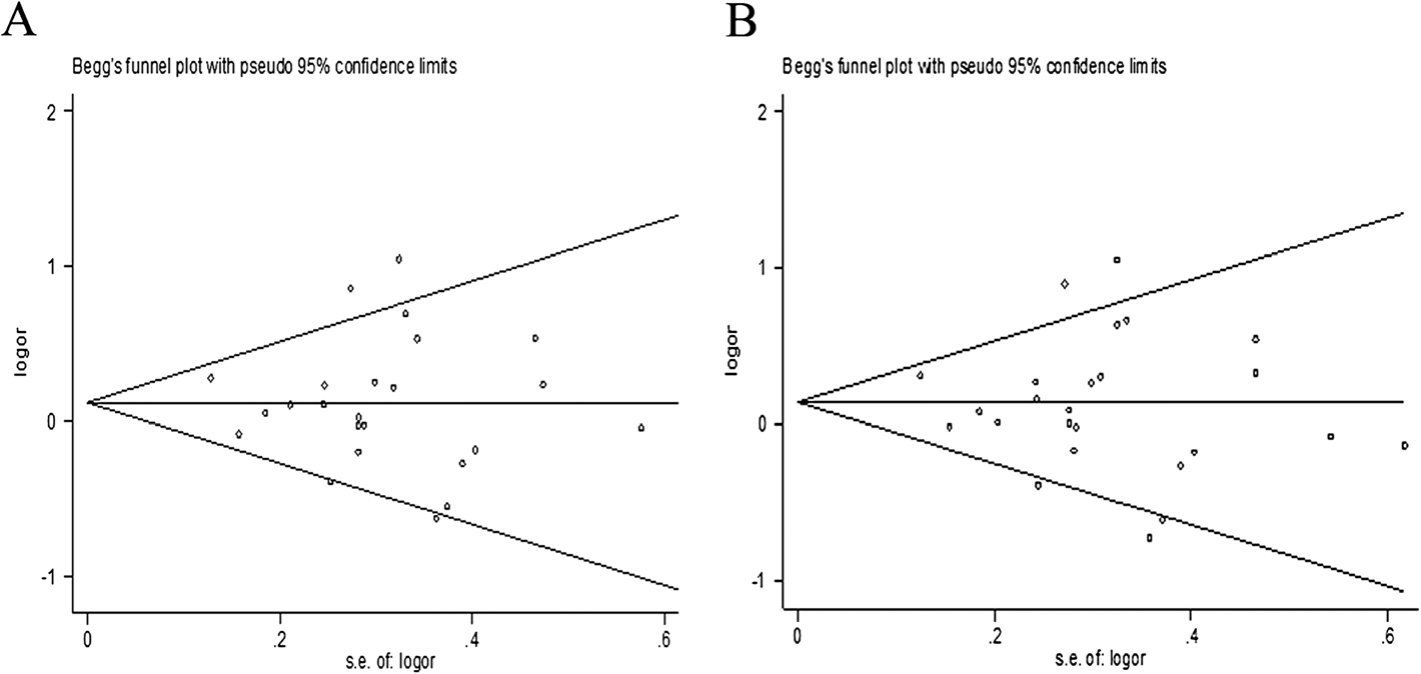
Funnel plot for publication bias of all eligible studies (A, CT vs. CC; B, CT + TT vs. CC).

There was no visible publication bias among the studies because of the shape of the Begg’s funnel plots revealed symmetry in the CT vs. CC and CT + TT vs. CC comparative genetic models (Figure 3). Statistical evidence of funnel plot symmetry was provided by Egger’s test. The results also showed no publication bias in the *C224T* polymorphism (t = -0.19, P = 0.853 for CT vs. CC; t = -0.34, P = 0.736 for CT + TT vs. CC).

## Discussion

The effects of genetic sequence variants in complex human traits are not readily detectable in population samples. However, meta-analysis that accumulates published data from small single research is a valuable tool in identifying disease genes. The functions of CTSD are to hydrolyse APP protein and clear Aβ from the central nervous system [10,11]. In AD patients, CTSD was expressed in the core of neuritic plaques [45], and cellular and cerebrospinal levels are elevated [46]. The variants of this gene might impede the proteolytic cleavage of APP and the degradation and clearance of Aβ, the synthesis of which is a supposed pivotal event in the pathogenesis of AD. Therefore, our motivation for the present study was to determine the association between *CTSD* polymorphism and AD risk from abundant data over 16,651 genotype cases and controls.

As far as we know, the present meta-analysis involving 5602 cases and 11,049 healthy controls was the most comprehensive to date to investigate the relation between the *CTSD C224T* polymorphism and AD susceptibility. Our finding indicated that the *C224T* polymorphism was not associated with the AD risk both in Asian and Caucasian populations, which were in accord with the results of the previous meta-analysis [38] and inconsistent with Schuur’s results [18]. Compared to the previous study, our meta-analysis has some particular strength. First, we had the largest sample size; we added four Asian population studies, the absence of which in the Schuur study might have caused a deviation in the final result; and ten new case–control studies were added compared to the Ntais study, which might have effectively altered the overall results. Second, because nearly half of the eligible studies did not detect the homozygous *TT* polymorphism, and the proportion of *TT* was very small, as is usual in common polymorphisms, heterozygote might be responsible for the significant difference in frequency; therefore, we only compared the CT vs. CC and the dominant CT + TT vs. CC models. Lastly, no significant publication bias was observed in any of the studies analyzing by Egger’s test and Begg’s funnel plot. Thus, based on the above factors, the results of our meta-analysis were more reliable than those of previous studies.

Our results from the CT vs. CC and dominant CT + TT vs. CC comparison models suggested that no significant correlation was existed between the *CTSD C224T* polymorphism and AD risk. Given that the control genotypes of two case-control studies [22,29] were out of HWE, they might have contributed some bias to our summary OR. When we excluded those two studies, the summary OR was not effectively altered, showing that our result was reliable. A great degree of heterogeneity among studies was identified for CT vs. CC (x^2^ = 39.65, P_Q_ = 0.023) and CT + TT vs. CC (x^2^ = 44.23, P_Q_ = 0.007) in the overall populations. Several factors might contribute to the heterogeneity. First, AD is a complicated and multi-genetic disease. Second, clinical heterogeneity, such as gender, age of onset, and diagnosis criteria, were factors. The different studied populations, such as ethnicity, might also explain the discrepancy. In subgroup analysis stratified by ethnicity and age of onset, heterogeneity only existed in the Caucasian subgroup, indicating that age was the major contributor to the existence of all heterogeneity.

Considering the impact on the summary OR of different ethnicities, we further performed subgroup analysis based on ethnicity. Those results indicated no significant association between the *CTSD C224T* polymorphism and AD risk either in Asian or in Caucasian population, which was inconsistent with the previous meta-analysis[18]. Similarly, the results did not change when the two studies that violated HWE [22,29] were excluded. The number of samples in the Asian subgroup was dramatically less than those in the Caucasian subgroup, which may weaken the conclusions. Our results also differed from the Schurr study after excluding the Mateo study in Caucasian population. While after excluding Albayrak [39] and Mateo [33] study, a significant association was found in the dominant CT + TT vs. CC genetic model(OR = 1.201, 95% CI = 1.004-1.436, P = 0.045). The principal cause for the difference with our results was the inclusion of the Albayrak [39] study. The Albayrak study reported that the *CTSD C224T* polymorphism increased AD risk in men only which might cause the false-negative result. As no study has clarified gender-specific differences regarding lysosomes or its components and the characteristic lesions in AD, therefore, future study with larger samples to investigate the gender-specific is necessary. When stratified by age of onset, we found no significant differences both in EOAD and LOAD subsets. Possible explanations for these findings might be the small sample sizes for analysis; the same control source, without strict age matching, and missing age information in some studies. Given these factors may affect the statistical power. Further research is required to assess the gene effects and validate our findings.

To evaluate the interaction of *CTSD* polymorphism and *APOEϵ4* allele on AD, ten studies which provided genotype distribution data of *APOEϵ4* status were chosen for further study, and of which only four showed evidence of an association [14,15,20,25]. The results of our study showed non-significant relation between the *C224T* polymorphism and AD risk in *APOEϵ4* carriers and non-carriers. The association of *CTSD T* allele with AD risk between *APOEϵ4* carriers and non-carriers in Caucasians was quite similar, contrary to the Schuur result. Due to the lack of an Asian population in the Schuur study, sample size and ethnicity might have contributed to some bias in the final result. While the ORs of *APOEϵ4* were greater in the *T* allele carriers group than the subjects without the *T* allele. Because of the extensive overlap in two effect sizes and the remarkably small group of subjects who carry both the *APOEϵ4* and *CTSD T* alleles, the association between the *CTSD T* and *APOEϵ4* alleles should be interpreted cautiously.

There were some limitations that merit attention. First, some of the eligible studies lacked sufficient information for detailed and deep analysis. In some studies, the controls were not uniformly defined as matched by age and gender; and it may lead to some negative correlation. Second, we mainly focused on the *C224T* polymorphism, discounted the potential linkage disequilibrium with another mutation of this gene, and ignored the interactions between gene and gene or gene and environment. Third, the data of our meta-analysis was unadjusted; the suspected factors could be analysed, such as, gender, diet, lifestyle habit, and environmental factors. Fourth, we included the English or Chinese publications only; the lack of unpublished data and data published in other languages might contribute some bias. There were only four articles in the Asian subgroup, with small sample size, which may cause low statistical power.

## Conclusions

The finding of our present study revealed that the *CTSD C224T* polymorphism was not associated with AD risk both in the overall populations and the subgroups stratified by ethnicity and age of onset. In addition, we found no statistically significant differences between the *CTSD C224T* genotypes and AD stratified by *APOEϵ4* allele status. Our data did not suggest that the *CTSD C224T* polymorphism was a possible susceptibility factor for AD. Future studies will require much larger sample sizes and will need to analyse the impact of this polymorphism in other populations.

## Abbreviations

AD: Alzheimer’s disease; CTSD: Cathepsin D; APOE: Apollipoprotein E; EOAD: Early-onset AD; LOAD: Late-onset AD.

## Competing interests

All authors state no conflict of interest.

## Authors’ contributions

CM, QL, XQ, SL originally designed the study, collected the literature data and drafted the manuscript. JS, JW, YD performed data extraction and statistical analysis. LX, TL, YH executed literature search and checked the results. All authors have read and agreed with the final manuscript.

## Pre-publication history

The pre-publication history for this paper can be accessed here:

http://www.biomedcentral.com/1471-2377/14/13/prepub
